# Peer Support in Coordination of Physical Health and Mental Health Services for People With Lived Experience of a Serious Mental Illness

**DOI:** 10.3389/fpsyt.2020.00365

**Published:** 2020-05-08

**Authors:** Marianne Storm, Karen L. Fortuna, Jessica M. Brooks, Stephen J. Bartels

**Affiliations:** ^1^ Department of Public Health, Faculty of Health Sciences, University of Stavanger, Stavanger, Norway; ^2^ Department of Psychiatry, Dartmouth Centers for Health and Aging, Lebanon, NH, United States; ^3^ Department of Psychiatry, CDC Health Promotion Research Center at Dartmouth, Lebanon, NH, United States; ^4^ Columbia University School of Nursing, New York, NY, United States; ^5^ Mongan Institute, Massachusetts General Hospital, Harvard Medical School, Boston, MA, United States

**Keywords:** peer support specialist, coordination, serious mental illness, community mental health, medical health

## Abstract

**Background:**

Engaging peer support to augment and enhance traditional mental health services presents novel opportunities to improve service engagement and delivery. However, there has not been an in-depth analysis of the processes and methods behind the coordination of physical health and mental health care by peer support specialists.

**Study aim:**

To explore the potential of peer support specialists in community mental health centers and as a means to improve coordination of physical health and mental health services for people with a serious mental illness.

**Methods:**

We conducted 28 semi-structured qualitative interviews with peer support specialists and mental healthcare professionals in community mental health centers in two states (blinded for review) in the United States. Data were triangulated to explore peer support specialists and mental health professionals’ perspectives.

**Results:**

We found five themes characterizing the role of peer support services in the coordination of physical health and mental health services for individuals with serious mental illness: (1) Advocacy in interprofessional meetings, clinical teams, and advisory councils; (2) Sharing lived experiences and connecting with available resources and services; (3) Preparing for mental health and physical health care visits; (4) Mutuality; and (5) Affiliations, funding, and sustainability of peer support services.

**Conclusion:**

This study suggests that peer support specialists can uniquely contribute to the coordination of physical health and mental health services for individuals with serious mental illness.

## Introduction

People with serious mental illness (SMI) are challenged by numerous treatment and care needs in the context of a fragmented system of social, medical, and mental health services. Coordination of services is essential to meet the complex needs of individuals with SMI (1). In addition to managing mental health symptoms, individuals with SMI face high rates of medical comorbidity [i.e., heart disease, diabetes mellitus, hypertension, chronic asthma, and emphysema) (2, 3) and experience difficulties with managing these conditions (4). Coordination is a system of linking clinical and non-clinical services for individuals with SMI and includes the processes, measures, and networks at the interfaces of the mental health hospital, primary care delivery, and community health systems (1, 5)]. The U.S. Agency for Health Care Research and Quality (AHRQ) (6) refers to coordination as the organization of care activities between participants involved in a person’s care to facilitate the appropriate delivery of services. AHRQ has developed a framework that includes key coordination components, establishment of shared accountability, communication among involved stakeholders, exchange of information, facilitation of care transitions, clinical assessment of care needs and goals, careful monitoring and response to change, support of self-management, and providing links to community resources. Exchange of information, facilitating care transitions, supporting self-management, and providing links to community resources are coordination components in which a peer support specialist can uniquely contribute to coordinated physical health and mental health care for individuals with SMI.

Peer support services located in mental health settings are based on principles of respect, shared responsibility, and mutual agreement on what is helpful between the peer support specialist and a person with SMI (7). A peer support specialist has lived experience and is trained and accredited to provide emotional, social, and recovery support to individuals with similar mental health conditions (8). Sharing similar lived experiences can improve quality of life, reduce loneliness, enhance symptom control, and increase engagement in treatment for individuals with SMI (9–11). Peer support specialists can also encourage health behavior change in people with SMI (12–14) and can be valuable in the coordination of care transitions from inpatient units to a community setting (10, 15). Peer support specialists have been shown to help individuals with SMI to improve their self-management skills and to improve access to primary care (4). Peer support specialists are also being hired into traditional positions in mental health service organizations as members of integrated physical health and psychiatric teams (7, 16). While peer support has been associated with a variety of benefits, little is known about how peer support services can facilitate the coordination of physical health and mental health services for individuals with SMI. This study aims to describe the processes and methods behind peer support services in community mental health centers and the coordination of physical health and mental health services from the perspectives of mental health professionals and peer support specialists in two (blinded for review) states in the United States.

## Methods

We conducted a qualitative study that included individual key informant interviews (17). We interviewed a convenience sample (18) of mental health professionals working with individuals with SMI in community mental health centers. We used snowball sampling (19) to select interviewees with peer support specialists and include information-rich key informants.

### Ethics and Recruitment

The (blinded for review) Institutional review board approved this study. Interview participants were included based on their interest and willingness to participate in the study. Mental health professionals were recruited from six community mental health centers across two (blinded for review) states in the United States. For recruitment, the first author (MS) contacted the directors of the community mental health centers *via* email with information about the project. Upon agreement to include their organization in the study, interview participants were identified in each community mental health center to meet the inclusion criteria of being a mental health professional employed as one of the following: (1) administrative leader, (2) program manager, (3) clinician (i.e., psychiatrists, psychologists, social workers), or (4) case manager (i.e., individuals assigned the role of outreach case management and/or care coordination). Next, potential individual interview participants were contacted by MS *via* email and were included if they agreed to participate in an interview. MS performed all interviews with mental health service providers in person or over the telephone.

The second author (KF) recruited peer support specialists. They were recruited based on their experience in providing peer support services to individuals with SMI. Peer support specialists were contacted *via* email by KF. The email message contained information about the research project and an inquiry regarding potential interest in participating in an individual interview. Upon agreement to participate, peer support specialists were contacted *via* email to schedule time for a telephone interview. MS conducted three individual interviews with peer support specialists, while KF conducted two interviews.

All the interview participants were provided with written information about the research project and their right to privacy, that is, that the information collected would remain confidential and that names and other identifying information would not be used in any written paper. A gift card of $25 was provided in person or by postal mail after each interview to be used by the study participant or to be given to a service user.

### Data Collection

We conducted 28 individual qualitative interviews, including 23 interviews with mental health professionals employed in community mental health centers and five interviews with peer support specialists. Interview participants from community mental health centers included three center directors, five-division directors, four clinical therapists, five program leaders, and six clinical case managers. The participating peer support specialists provided services in peer-run programs and in Medicaid-reimbursable peer support services based in community mental health centers. We conducted interviews between January 2018 and June 2018. Ten interviews were performed in person by MS. The majority of the interview participants were female (n = 24). We interviewed 18 female mental health professionals and five female peer support specialists. The interviews lasted between 20 and 60 min (average 30 min). All interviews were audio-recorded.

We used a semi-structured interview guide to explore mental health professionals’ and peer support specialists’ views of peer support services in community mental health centers and the coordination of physical health and mental health services for individuals with SMI. The semi-structured interview guides were related to the coordination activities as identified by AHRQ (6) and included questions about coordination of needed care (i.e., physical health and mental health care needs) and coordination with other sectors (i.e., nursing homes, home care services, housing, and vocational and other social services). There were also specific questions about peer support services (e.g., the role of peer support specialists in the community mental health center, what services they provide, and how peer support services are funded).

### Data Analysis

We transcribed the audiotaped interviews into written transcripts. For an overall understanding of the data, MS developed an initial structure comprising codes derived from the coordination activities in the AHRQ measurement framework (6). Thematic analysis was used to identify patterns in the data (20). MS coded all the interview transcripts through a process of reading the transcripts, identifying and applying codes to the text, and collating the codes into potential themes documenting patterns in the data (20). To minimize bias and increase transparency in the analysis, KF coded 10 interviews, 5 interviews with mental health professionals, and all interviews with peer support specialists in a similar process as MS. All the potential themes were then checked by MS in relation to the data material, including refining specifics and naming of each theme. The final set of summary themes were based on a consensus among the study’s authors.

## Results

The data analysis produced five themes: (1) Advocacy in interprofessional meetings, clinical teams, and advisory councils; (2) Sharing lived experiences and connecting with available resources and services; (3) Preparing for mental health and physical health care visits; (4) Mutuality; and (5) Affiliations, funding, and sustainability of peer support services. The themes provide a description of the role of peer support services in community mental health centers and the coordination of physical health and mental health services for individuals with SMI.

1. Advocacy in Interprofessional Meetings, Clinical Teams, and Advisory Councils
  Peer support specialists can take part in weekly residential meetings or advocate in interprofessional team meetings if the service user approves. A case manager said, *“Sometimes we have a team meeting for different clients and the peer support person was there. I had a team meeting the other week because I had a client who just was upset about something her therapist had done. And the peer support person was there to add her insight”.*
  The peer support specialist can support the individual by making sure that he or she is represented and heard, including in situations where he or she does not want to participate. For example, a peer support specialist can attend a meeting with mental health professionals on behalf of a service user. Illustrative of this is the following quote from a peer support specialist: *“So whether that means to have the person write down and meeting for them when they don’t feel comfortable and then making sure that their team knows, that these are her words, and this is her thoughts. And then ideally it’s a progression to be where the person can speak up and have her/his voice and doesn’t need that extra”.*
  In some of the community mental health centers, peer support specialists serve as members on clinical teams such as assertive community treatment teams (ACT teams) in the community and mobile crisis teams. A director of community support services said, *“We do have an allocation of peer specialists directly on the clinical teams, especially on the ACT teams and they are also part of that process in terms of trying to link the service user up to necessary community-based services”*).
  According to mental health professionals, peer support specialists may serve as members of the agency’s board of directors or they sit on a separate “peer council/peer advisory board” that meets regularly with representatives from the community mental health center’s agency board of trustees and staff members. Hence, peer support specialists participate and have a voice in agency policy discussions about service coordination.
  Professionals across the community mental health centers emphasized the importance of the perspective of people with lived experiences for challenging the views of clinicians and mental health professionals and shift the power balance within the mental health system. An illustrative quote from a clinical therapist in a community mental health center was, “*The perspectives of people with lived experiences at every table so that it keeps us (clinicians) check.*”
2. Sharing Lived Experiences and Connecting With Available Resources and Services
  According to the mental health professionals, peer support specialists in the community mental health centers provide practical and emotional support by sharing lived experiences and their strategies for self-management.
  A clinical therapist said, *“They are able to talk to people. If people really want to go off their meds, their ability to talk to them about that and about the pros and cons, if you want to do it and how you do it.”*
  In several of the community mental health centers, peer support specialists lead weekly support groups where they talk about advocacy; social justice; and how to navigate the mental health, social, and primary care service systems. A division director said, “*We have what’s called a Life enrichment center, which is a peer-run center here in this office, that have peer support group offerings. They talk a lot about advocacy and social justice and navigating the system. They talk a lot about alternatives to the traditional medical model of mental health treatment*”. Mental health professionals elaborated that they believe peer support specialists are helpful because of what they can provide from the perspective of lived experience. They often have connections with networks in the community that can be helpful to the individual. They can provide follow-up contacts; go with the person to appointments in the mental health, primary care, or social service system; and act as advocates during meetings.
  One peer support specialist talked about how she provided peer support *via* text messages emphasizing that texting could be beneficial when the person does not want to talk on the phone or see her in person. The peer support specialist said, “*they rather text it out.*” Another peer talked about wellness and promoting healthy living habits. She said “*I think peer can be very powerful coaches for individuals who want to live healthier*”.
3. Preparing for Mental Health and Physical Health Care Visits
  There were examples of programs that included peer support specialists to help individuals with SMI in preparing for physical health and mental health care visits. Several interviewees mentioned a web-based program called CommonGround, which is a collection of tools supporting recovery and healing after a diagnosis of mental illness (21). Peer support specialists take part in the CommonGround program to assist individuals with SMI in preparing for their upcoming appointment with the doctor.
  One licensed mental health counselor said, *“We have three peer support specialists that work here at the center. They have a kind of specific role right now, which is helping people. They meet with all the clients prior to their prescribed appointment to help them fill out kind of a questionnaire. It’s like an internal questionnaire online about their mental health status, basically and any changes since their last appointment. So, the peer support specialists help people through that process prior to their prescribed appointment and we use a system call CommonGround for that”.*
  According to the interviewees, the CommonGround program includes an overview of the person’s five last visits to the community mental health centers and an overview of how things have been going between the visits (for example, how the person has been sleeping). A peer support specialist said, “*The CommonGround helps folks to prepare for their appointments and to be more in charge of their appointments with their physician and to speak up for what they need.*”
  Another program referred by mental health professionals included peer support specialists to support individuals with SMI when they are in a crisis, for example, when they are in the hospital emergency rooms while waiting for a hospital bed. A program director said, “*They offer support to people who are boarding in the hospital emergency rooms. So, if somebody needs hospitalization and there’s not a bed, they can wait in the hospital emergency room. And so, they provide advocacy and support and help people understand the system and help them tolerate being in the emergency room*”.
4. Mutuality
  The peer support specialists were conscious that their role was distinct from the mental health professional clinical role and that they were in a mutual relationship with each service user. One peer support specialist said, “*Our role is very, very different from that of professionals. We emphasize respect for the individual and mutuality between peer counselor and counselee.*” The peer support specialists were particularly aware of the need to not take on clinical tasks that could put them in a position that would enable them to control someone’s behavior and reduce the mutuality in the relationship with the individual. For example, this could happen in situations associated with forced compliance with medications, the assertion of control over the individual, and are in conflict with the peer code of ethics. One peer support specialist explained that some agencies do not want peers to engage in discussions about medications at all. Meanwhile, others leave it up to the peer support specialist. Two peer support specialists explained how they would deal with the issue of medications: “*I can’t recommend medication, because I’m not a doctor, but I can definitively sit in the office and help you advocate about your symptoms and what you want to do moving forward.*” The other peer support specialist said “*I try to stay away from having any kind of a professional or medicalized role and so i pretty much defer to the treaters about issues related to psychiatric medications*”.
5. Affiliations, Funding, and Sustainability of Peer Support Services
  In some of the community mental health centers, peer support specialists were affiliated with statewide peer support programs that have a contract with the community mental health center; such programs include the National Alliance on Mental Illness (NAMI), the Psychiatric Survivor group, and the Bipolar Depression Peer Support group. The peer support specialists receive hourly pay for their work at the community mental health center. Other community mental health centers have hired peer support specialists in positions on clinical teams (i.e., mobile crisis response teams, ACT teams) providing them with insurance, paid time off, and the ability to go to training. Peer support specialists were also funded through grants and fundraising. One clinical therapist said, “*Peer support specialists can receive stipends through for example NAMI, but this is not the same as salaried employment as it does not allow them to earn social security.*”
  Peers support specialists that are hired have gone through a certification process. Some also receive training and supervision at the community mental health center and from statewide peer support groups. According to mental health professionals, a few centers also organize peer support supervision. Peer support supervision was illustrated in the following comment from a rehabilitation team manager, “*We have a day program which they support and offer intensive training around peer support. We also do co-reflection, which is peer supervision once a month*”.
  Several mental health professionals addressed the difficulties of sustaining and maintaining the same group of peer support specialists over time. The difficulties were related to problems with sustaining project funding for peer support services, and the difficulties the peer support specialist could experience in managing their health issues. One case manager remarked, “*We have had a number of peer specialists that have started with us and none of them have lasted for more than a couple of months.* W*e’ve had difficulty having peer specialists come in and be able to manage both their health issues plus issues of being a team person working with us.*”
  A few peer support specialists observed that it could be stressful to bring experiences from work home because they care about the person. One peer support specialist said, “*You build a connection and you try to keep it separate from our own life and it can be a challenge for most people. I do have a psychologist as a therapist to help me separate my life from my professional life.*”

## Discussion

This study reviewed the processes of peer support in community mental health centers and peer support coordination of physical health and mental health services for individuals with SMI across two (blinded for review) states. The study identified five themes: Peer support specialists take on multiple roles in the community mental health centers in which they can advocate and have input to the coordination of physical health and mental health services. Peer support specialists share their experiences to connect the person with needed services. In some programs, the peer support specialist assists the individual in preparing for mental health and physical health care visits. Peer support specialists emphasize mutuality in their relationship with service users. Affiliations, funding, and sustainability of peer support services vary across the community mental health centers and impact the peer support specialist role in coordinating physical health and mental health services.

In the past few decades, peer support was predominantly available only in peer-run organizations and self-help groups, but peer support services are now being offered as group programs or one-to-one services integrated within inpatient, outpatient, and community-based clinical settings across the globe (7). Our results show that peer support specialists have physical health and mental health coordination capacity. They participate in interprofessional team meetings and they may function as members of the clinical ACT team or mobile crisis team. Peer support specialists also assist individuals in preparing for upcoming visits with health professionals. Incorporating lived experiences into service delivery in clinical settings has the potential to contribute to culture change, inspire hope, promote an equal relationship between service users and professionals, and facilitate service redesign (8, 22, 23).

Study results show that peer support specialists value mutuality in the relationship with the service user in coordination situations. However, a peer support specialist and a mental health professional have distinct roles in coordinating the person’s physical health and mental health services. This aligns with the professional practice standards for peer support specialists in the United States (24), which include ethical guidelines for peer support practice (i.e., peer support is mutual and reciprocal and involves equally shared power). In working with a peer support specialist, it is therefore important to recognize the need to align the role with peers’ ethics and values. Our results show that peer support specialists use their own experiences to help coordinate and connect service users with services in the community. Peer support specialists on coordination teams can foster a better understanding of a person’s mental health and physical health conditions by mental health professionals and can reduce power imbalances within the service system. These are important steps in developing organizational and cultural norms inclusive of service users, and peer support services in clinical settings that will maximize the benefits of peer partnerships in clinical settings and fulfill policy expectations (7, 22).

Solomon (8) points out that peer support specialists can experience personal and professional growth, improve their ability to cope with their mental health conditions, experience a sense of empowerment, and build their job skills. Research studies report that peer support specialists can experience difficulties with managing their role, low payment, and job insecurity, job stress that influences their wellness, and a lack of support from managers and clinical staff (25, 26). Formal and informal support and supervision of peer support specialists, better pay, recovery-focused organizations, and training may help to support individual resilience and sustainability (25, 27).

Research studies have reported that peer support can improve the transitions from inpatient psychiatric units to community settings (10, 15) and improve access to primary care for people with SMI (12). Potentially, peer support specialists can have a role in health behavior change and chronic disease self-management for individuals with SMI and chronic conditions (4). For example, a digital mental health intervention developed by our group called “PeerTech” combines peer and technology-based physical health and mental illness self-management intervention. Preliminary pilot study findings suggest a positive influence on health behavioral change (28, 29). Facilitators for successful integration of peer support services in clinical settings (22) include role clarity; adequate and reliable funding; and involvement and support from leaders, managers, and clinical staff members in the process.

There are some important limitations of this study that needs to be considered in interpreting the findings. First, the study consisted of a small sample of peer support specialists recruited using snowball sampling, as we were not able to recruit peer support specialists and mental health professionals from the same community mental health centers. Second, our study did not include the perspective of service users that could have had important information to add about how peer support specialists have influenced on the coordination of services. Third, although all interviews with mental health professionals were conducted by MS in-person or over the phone, two interviewers were conducting the interviews with the peer support specialists. Having different interviewers could have influenced the interview situations and the data collected.

Although our ability to generalize the study results is limited, we do believe that the results provide relevant and new knowledge about peer support specialists in community mental health centers and the coordination of physical health and mental health services. Among the strengths of the current study is the inclusion of mental health professionals from six community mental health centers across two states, and peer support specialists from both peer-run programs and peer support services in community mental health centers. Besides, our inclusion of mental health professionals and peer support specialists facilitated the acquisition of different perspectives on the role of peer support in mental health and physical health care coordination.

## Conclusion

This study contributes to current knowledge of peer support services in community mental health centers and how peer support specialists can support the coordination of physical health and mental health services for individuals with SMI in two (blinded for review) states. Future research is warranted to evaluate the effectiveness of co-designed peer support in the coordination of physical health and mental health services.

## Data Availability Statement

The datasets generated for this study are available on request to the corresponding author.

## Ethics Statement

The studies involving human participants were reviewed and approved by Trustees of Dartmouth College Dartmouth-Hitchcock Medical Center Committee for the Protection of Human Subjects. The patients/participants provided their written informed consent to participate in this study.

## Author Contributions

MS designed the study, carried out the data collection, performed data analysis, and prepared the manuscript. KF contributed to the study design, took part in data collection, and contributed to the data analysis and drafting of the manuscript. JB contributed with comments and drafting of the manuscript. SB contributed to the study design and drafting of the manuscript. All authors read and approved the final manuscript.

## Funding

First author MS received support for this research by the Norwegian Research Council Grant Agreement No. 276638 and the Commonwealth Fund. The views presented here are those of the authors and should not be attributed to the Commonwealth Fund or its directors, officers, or staff. The second author KF was funded by a K01 award from the National Institute of Mental Health (K01MH117496).

## Conflict of Interest

The authors declare that the research was conducted in the absence of any commercial or financial relationships that could be construed as a potential conflict of interest.
